# Modelling the genetic aetiology of complex disease: human–mouse conservation of noncoding features and disease-associated loci

**DOI:** 10.1098/rsbl.2021.0630

**Published:** 2022-03-23

**Authors:** George Powell, Helen Long, Louisa Zolkiewski, Rebecca Dumbell, Ann-Marie Mallon, Cecilia M. Lindgren, Michelle M. Simon

**Affiliations:** ^1^ Big Data Institute, Li Ka Shing Centre for Health Information and Discovery, University of Oxford, Oxford OX3 7LF, UK; ^2^ MRC Harwell Institute, Mammalian Genetics Unit, Oxfordshire OX11 0RD, UK; ^3^ Department of Physiology, Anatomy and Genetics, University of Oxford, Oxford OX3 7BN, UK; ^4^ Wellcome Centre for Human Genetics, University of Oxford, Oxford OX3 7BN, UK; ^5^ Nottingham Trent University, Clifton Lane, Nottingham NG11 8NS, UK; ^6^ Medical and Population Genetics Program, Broad Institute of MIT and Harvard, Cambridge, MA 02142, USA

**Keywords:** complex disease, Mendelian disease, orthologue, alignment, annotation, conservation

## Abstract

Understanding the genetic aetiology of loci associated with a disease is crucial for developing preventative measures and effective treatments. Mouse models are used extensively to understand human pathobiology and mechanistic functions of disease-associated loci. However, the utility of mouse models is limited in part by evolutionary divergence in transcription regulation for pathways of interest. Here, we summarize the alignment of genomic (exonic and multi-cell regulatory) annotations alongside Mendelian and complex disease-associated variant sites between humans and mice. Our results highlight the importance of understanding evolutionary divergence in transcription regulation when interpreting functional studies using mice as models for human disease variants.

## 1. Background

Understanding the mechanistic function of disease-associated loci is a fundamental challenge for biomedical research, and is critical for the development of effective treatments and drug targets [1]. Genome-Wide Association Studies (GWAS) have identified a myriad of variant sites associated with the risk of complex diseases [2]; however, the causal pathways of these loci remain poorly understood [3]. This is in part due to the relative difficulty of functional follow-up studies, which is compounded by the small and potentially interactive effects of variants, and the complexity of interpreting the function of non-coding regions, where the majority of GWAS variants are found [4].

The mouse is the most commonly used mammalian model for biomedical research [5–8] and has been used to infer the function of human disease variants. Mouse models have been particularly useful for elucidating the function of variants in protein-coding transcripts, which are highly conserved between the species [9], in addition to loci associated with traits that can only be measured *in vivo* such as body fat distribution or body mass index [10]. The mouse is also the only non-human mammal for which we have data on regulatory feature occupancy from genomic assays catalogued by ENCODE [11,12]. It is therefore uniquely suited to serve as a model for understanding regulatory feature function, with further potential for modelling human disease loci through humanization of the mouse genome using CRISPR/Cas9 technologies [5,13,14]. Studies have mapped human GWAS variants associated with given disease phenotypes to the mouse genome and shown an enrichment in regions linked to transcription regulation [11,15–17]. Studies have also, however, highlighted the substantial divergence in tissue and/or cell-specific transcription regulation between the species [11,12,15,18–20], making it unclear in which instances the mouse can recapitulate mechanisms of human gene expression to sufficiently model the function of human disease-associated genetic variants [21].

The Ensembl Regulatory Build amalgamates datasets from various consortia, including ENCODE, to annotate predicted regulatory sequences across the human and mouse genomes [22]. These annotations are continually updated as more data become available and, importantly, have stable identifiers to provide a reference framework for ongoing research. It is, however, currently unreported how human–mouse alignment compares across the spectrum of annotation categories. Furthermore, it remains unclear how Mendelian and complex disease-associated variant site alignment varies between different regulatory annotations. Addressing these two questions would provide a useful reference point for researchers considering mouse models for human disease-associated loci.

Here, we use genomic annotation from Ensembl to provide a genome-wide overview of sequence alignment for twelve categories of annotation (including exonic and regulatory features) between humans and mice. We assess the alignment of Mendelian and complex disease-associated variant sites between the species across these annotation categories and discuss the implications of our results for the use of mouse models to understand the mechanistic function of human disease loci.

## 2. Results and discussion

**Figure 1.**
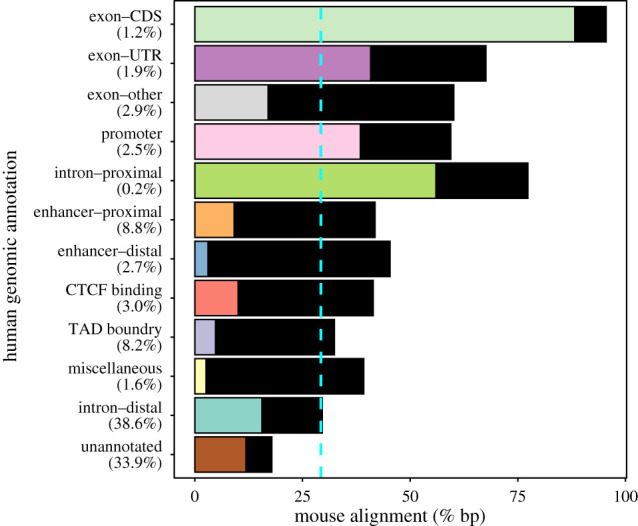
Alignment of genomic annotations between humans and mice. Bars represent the percentage of human bases that align with the mouse genome. Coloured bars represent the percentage of bases that align with a common annotation in the mouse (i.e. the same annotation in each species). Black bars represent the percentage of bases that align to a different annotation in the mouse (i.e. do not have a common annotation). The dashed blue line represents the genome-wide percentage of human bases that align with the mouse genome. The genomic coverage for each human annotation is labelled in brackets on the *Y*-axis. The sum of coverage is greater than 100% due to the overlap of annotations (electronic supplementary material, table S2). Human protein-coding sequences show the greatest alignment to the mouse genome (95.5%). The fraction of human annotation that aligns to the same annotation in mice is highest for protein-coding sequences (88.2%), proximal intronic sequences (56.0%), untranslated regions (UTRs; 40.8%) and promoters (38.5%), and lowest for distal enhancers (3.1%), topologically associated domain (TAD) boundaries (4.8%) and miscellaneous (2.6%). CTCF, CCCTC‐binding factor, which is encoded by the *CTCF* gene; CDS, coding DNA sequence.

The human and mouse genomes have been annotated genome-wide by the Ensembl Regulatory Build [22] and GENCODE [23], and we used these two sources to annotate all base-pair positions across the autosomes for both species (see §4 and electronic supplementary material). Species genomes that have diverged over evolutionary time can be aligned to identify orthologous loci [24]. Throughout this manuscript, we define human bases as aligned if they have an orthologous base in the mouse genome (i.e. if they have a corresponding genomic position in the pairwise alignment conducted by Ensembl [25]), independent of whether a point mutation has occurred. We summarize the overall fraction of human bases that align to the mouse genome for each annotation category (figure 1). We describe the fraction of human bases with a given annotation that align to bases with the same annotation in the mouse genome as having common annotation.

In total, 29.3% of the human autosome aligns to the mouse; however, alignment varies by genomic annotation (figure 1; electronic supplementary material, table S3). Human translated exons (95.5%), proximal intronic sequences that include splice sites (77.3%), 3′ and 5′ untranslated regions (UTRs) (67.6%) and promoters (59.4%) show a relatively higher degree of alignment to the mouse genome than other exonic and regulatory annotations, including proximal and distal enhancers (41.8% and 45.3%, respectively), miscellaneous sequences (39.2%), CCCTC‐binding factor (CTCF) binding sites (41.4%) and topologically associated domain (TAD) boundaries (32.4%). The fraction of bases that align to the mouse and have common annotation in both species provides a coarse measure of feature conservation. The fraction of common annotation varies by annotation category and is greatest for translated exonic sequences (88.2%), followed by proximal intronic regions (56.0%), UTRs (40.8%), and promoters (38.5%) (figure 1; electronic supplementary material, table S3). By contrast, the fraction of common annotation is lower for proximal and distal enhancers (9.2% and 3.1% respectively) (figure 1; electronic supplementary material, table S3). This is consistent with previous research highlighting the rapid rate of enhancer turnover relative to promoters across mammalian species [7,26–29]. However, because enhancers can act as tissue-specific *cis*-regulatory elements [30], and the human and mouse regulatory builds are constructed using different amalgamations of tissue sources [22], some enhancer annotation and alignment may not have been captured by the multicell annotation model. We assessed the alignment of tissue-specific enhancers by comparing the alignment of enhancers active in adult heart, liver and spleen samples between the species (electronic supplementary material, table S4). Tissue-specific enhancers have a comparable alignment (ranging from 42.2% to 51.0% for proximal enhancers and 43.5% to 60.2% for distal enhancers) to the multicell model but were less conserved (ranging from 1.6% to 5.1% for proximal enhancers and 0.4% to 2.7% for distal enhancers).

**Figure 2.**
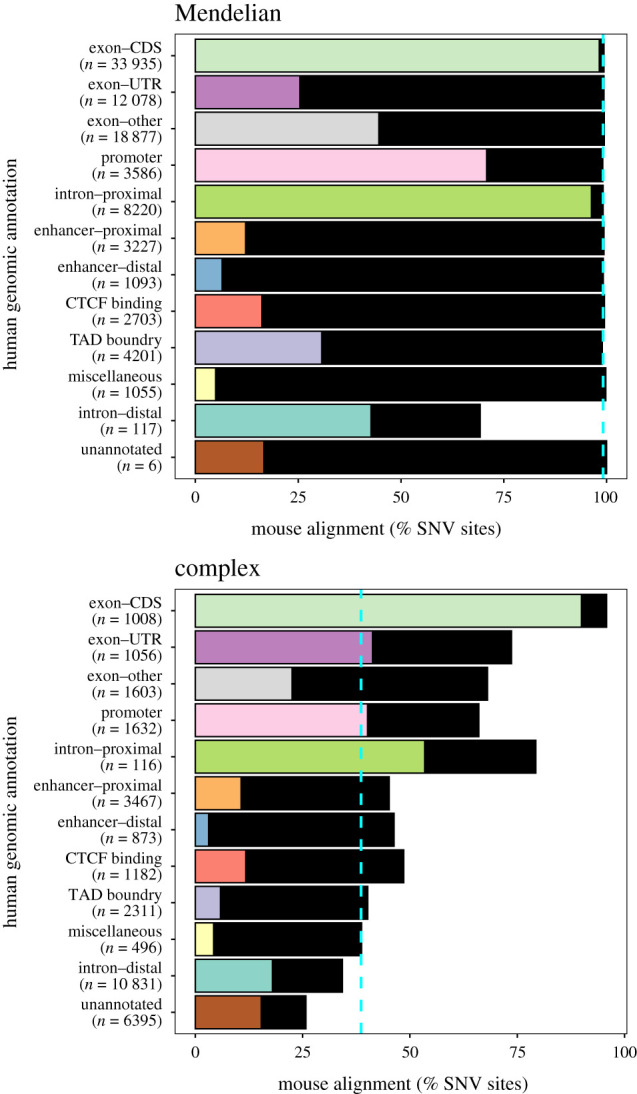
Alignment of human SNV sites associated with complex disease (GWAS Catalog) and Mendelian disease (ClinVar) between humans and mice. Bars represent the percentage of human variant sites that align with the mouse genome. Coloured bars represent the percentage of variant sites that aign with a common annotation in the mouse (i.e. the same annotation in each species). Black bars represent the percentage of variant sites that align with a different annotation in the mouse (i.e. do not have a common annotation). The dashed blue line represents the total percentage of variant sites that align with the mouse genome. Variant sites associated with human Mendelian disease are more conserved between the species than variant sites associated with human complex disease. However, annotation of non-exonic regulatory features (excluding the promoter) is poorly conserved, suggesting functional divergence between the species.

It is important to determine the similarities and differences in regulatory architecture between humans and mice when considering using a mouse model to infer the mechanistic function of human disease-associated variants [21]. We assessed the alignment of human variant sites predicted to cause Mendelian disease and human variant sites associated with complex disease with the mouse genome by considering two datasets: single nucleotide variant (SNV) sites predicted to cause Mendelian disease from ClinVar [31] (*n* = 42 039) and SNV sites associated with complex disease from the GWAS Catalog [32] (*n* = 27 794). As expected, both Mendelian and complex disease-associated variant sites in translated human sequences (Exoncoding DNA sequence (CDS)) have a high degree of alignment to the mouse genome (99.3% and 95.8%, respectively) (figure 2; electronic supplementary material, table S5). Across non-protein-coding sequences (i.e. loci not classified as ExonCDS, hereafter referred to as non-coding), 98.4% of pathogenic variant sites predicted to follow Mendelian inheritance patterns have an orthologous position in the mouse genome (figure 2; electronic supplementary material, table S5). This is significantly more than the genome-wide average of 28.8% for non-coding loci (*z* = 139.5, *p* < 1.0 × 10^−300^) and indicates that these sites have had a higher probability of being constrained by local purifying selection, potentially as a result of functional importance, since the species' divergence. There is, however, variation in the fraction of SNV sites that align to the same annotation in mouse between regulatory elements. 70.8% of Mendelian pathogenic SNV sites in human promoter sequences align to mouse promoter sequences. In comparison, only 12.2% of Mendelian pathogenic SNV sites in human proximal enhancers and 6.5% in human distal enhancers align to loci with the same annotation in mice (figure 2; electronic supplementary material, table S5). This difference suggests that while these loci may have had a higher probability of preservation due to local purifying selection in both lineages, the active regulatory elements and functional pathways at these variant sites have diverged. It must be noted, however, that some similarities may be missed due to regulatory feature specificity and differences in the tissue amalgamations used to annotate regulatory features.

A significantly smaller fraction of non-coding variant sites associated with complex disease aligns with the mouse genome than non-coding variant sites predicted to follow Mendelian inheritance patterns (36.4% compared with 98.4%, *z* = 97.7, *p* < 1.0 × 10^−300^) (figure 2; electronic supplementary material, tables S5 and S6). One explanation for this may be the small effect size of variant sites associated with complex disease having limited fitness effects [33]. Distal introns and unannotated regions contain the majority (62.0%) of variant sites associated with complex disease, making their effect on transcription regulation difficult to infer. However, a significantly greater fraction of variant sites associated with complex disease in these regions aligns with the mouse genome than the total fraction of bases with these annotations: 34.3% compared with 29.6% (*z* = 10.7, *p* = 9.10 × 10^−27^) for distal introns and 25.8% compared with 17.9% (*z* = 16.5, *p* < 4.90 × 10^−61^) for unannotated (electronic supplementary material, table S8). This suggests that the functional role of loci within regions annotated as ‘intron–distal’ and ‘unannotated’ has not been captured by the annotation model and may discourage the production of mouse models for these variants.

## 3. Conclusion

By comparing the mouse and human genomes, we found that 95.5% of human protein-coding sequence and 28.5% of human non-coding (untranslated) sequence aligns with the mouse genome. Furthermore, 98.4% of human non-coding variant sites associated with Mendelian disease align to the mouse genome, compared with 36.4% of non-coding variant sites associated with complex disease. The degree of overall divergence in the regulatory landscape between humans and mice highlights the importance of understanding the differences between functional pathways of interest when using mouse models to infer human disease mechanisms.

## 4. Methods

Regional genomic annotations for human and mouse autosomes are defined by the Ensembl Multicell Regulatory Build [22] and GENCODE [23] from Ensembl (v.101) [34]. Exonic genomic regions were categorized by their GENCODE annotations as: ‘exon-CDS’ for translated nucleotides in protein-coding exons; ‘exon-UTR’ for 5′ untranslated region (UTR) or 3′ UTR nucleotides in protein-coding exons; ‘exon-other’ for nucleotides in non-protein-coding exons (notably ncRNAs and lncRNAs). Regulatory regions were categorized by their Ensembl Regulatory Build annotations as: ‘promoter’, ‘enhancer-proximal’, ‘enhancer-distal’, ‘CTCF binding site’ or ‘miscellaneous’ for nucleotides categorized as unannotated transcription factor binding site or unannotated open chromatin. Intronic nucleotides in either protein-coding or non-protein-coding genes were inferred from exon coordinates as annotated in GENCODE, and categorized as either: ‘Intron-proximal’ if they are located within 10 bp of a splice-site position, or ‘Intron-distal’ if they are located more than 10 bp from a splice-site position and do not have any other annotation. TADs were called using the Arrowhead algorithm [35] (detail provided in the electronic supplementary information) and TAD boundaries were defined as ±25 kb from the start and end of each called TAD. All remaining nucleotides not annotated in GENCODE, the Ensembl Regulatory Build or as intronic are categorized as ‘Unannotated’. A summary of the genomic coverage for each annotation is provided in electronic supplementary material, table S1. Annotation overlap is summarized in electronic supplementary material, figure S1 and table S2. Human–mouse pairwise alignment was conducted by Ensembl (v. 101) using LastZ [24,25]. Human single nucleotide variant (SNV) sites associated with Mendelian disease were downloaded from ClinVar [31]. We considered all SNV sites with clinical significance labelled as either ‘Pathogenic’ or ‘Likely pathogenic’, and a review status labelled as either ‘criteria provided, multiple submitters, no conflicts', ‘criteria provided, single submitter’, or ‘reviewed by expert panel’ (*n* = 42 039). Human SNV sites associated with complex disease were obtained from the GWAS Catalog [32] and have a phenotype that is ontologically classified as either disease, disorder or cancer, and a *p*-value < 10^−8^ (*n* = 27 794). We tested differences in proportions using two-proportion *z*-tests (more information provided in the electronic supplementary material). All analysis and figure plotting were conducted in R v. 3.4.2 [36]. Detailed methodology is provided in the electronic supplementary material.

## Data Availability

The datasets supporting this article are publicly available and are described in the manuscript and/or electronic supplementary material. Data and custom scripts for analysis and figure generation can be found at: https://datadryad.org/stash/share/Odj-AMmF9W4irVVNJnPJ08nBsCoB6DEXKisohJ39XBc. https://doi.org/10.5061/dryad.8pk0p2nq5.
